# Supplementary material for: Divergent national-scale trends of microbial and animal biodiversity revealed across diverse temperate soil ecosystems
Source: Nat Commun. 2019 Mar 7;10:1107. doi: 10.1038/s41467-019-09031-1 (PMC6405921; doi:10.1038/s41467-019-09031-1)
Supplement: Supplementary file 1 — Supplementary Information [file 41467_2019_9031_MOESM1_ESM.pdf]

Divergent national-scale trends of microbial and animal biodiversity revealed across diverse temperate soil ecosystems

George, et al.

Supplementary Material

Supplementary Notes

1. Creation of Aggregate Vegetation Classes

The land use classification used in this study was originally developed for the UK Countryside Survey in 1990. In short, vegetation data was collected from 508 1 km randomly selected squares across the UK. Within each square, vegetation was recoreded in a number of plots placed either placed randomly or targeted to cover semi-natural habitats and along various landscape features such as field boundaries, hedges, and roads. This vegetation data was grouped into 100 vegetation classes using the TWINSPAN programme[1]. Then, detrended correspondence analysis using DECORANA[2] clustered these 100 vegetation classes into 8 Aggregate Vegetation Classes (AVCs), of which 7 were identified in the current study (Supplementary Table 1). The AVCs are ordered according to soil nutrient content[3], from the high-nutrient crops to the low-nutrient bogs, the order is listed in Supplementary Table 1. Such a decline in soil nutrient content also implies both productivity and management intensity gradients.

2. Glastir Monitoring and Evaluation Programme

The Glastir Monitoring and Evaluation Programme (GMEP) has been designed to assess the outcomes of implementing the Welsh Government's Glastir agri-environment scheme. GMEP is a collaboration funded by the Welsh Government and the European Union. The GMEP programme is run by the NERC Centre for Ecology and Hydrology and is a collaboration between specialists from public research centres, universities, voluntary bodies, and consultancies. When active, GMEP was the largest and most in-depth monitoring programme measuring environmental state and change within the European Union[4]. GMEP follows a holistic ecosystem approach with a rolling annual

survey conducted across areas both participating in and abstaining from Glastir. The results of the field survey were combined with national data and models to produce findings that inform stakeholders. A final summary of GMEP has been been published and is accessible to the public[4].

3. Soil maps in Wales

The soils at each sampling point were assigned to soil type using the National Soil Map and Soil Classification[5]. This map and classification scheme is derived from Avery[6] with revisions from Clayden and Hollis[7]. Soils were assigned to groups based on published soil maps and reconnaissance mapping of previously unsurveyed sites (for more detail see Cranfield University[5]). Generally, soils in Wales are known to map poorly, however, due to the high level of local heterogeneity. As such we found that the soil type factor was often dropped from linear mixed-models used to assess richness of soil organisms across land uses.

4. Pair-wise comparisons of bacterial, fungal, and archaeal richness

Linear mixed-models showed significant trends across land use types. Bacterial richness decreased ($F_{6, 264} = 78.47$, $p < 0.0001$) in AVCs across the productivity gradient, with highest values in the most productive Crops/weeds and grasslands and lowest in the low productivity land uses (Fig 3a). Specifically, richness in Heath/bog sites was significantly lower than all other AVCs except Upland wood ($p = 0.003$ for Moorland grass-mosaic; $p = 0.002$ for Lowland wood; $p < 0.0001$ for rest). Richness in the Crops/weeds, Fertile grassland, and Infertile grassland AVCs was also significantly greater than Upland wood ($p = 0.01$ for Crops/weeds; $p < 0.001$ for both grasslands). Additionally, higher levels of bacterial OTU richness were observed in Fertile grassland and Infertile grassland AVCs

when compared to both Lowland wood ($p = 0.002$; $p < 0.001$, respectively) and Moorland grass-mosaic sites ($p < 0.001$ for both).

Fungi ($F_{6, 248} = 48.98$, $p < 0.001$; Fig. 3b) and protists ($F_{6, 249} = 59.86$, $p < 0.001$; Fig. 3c) followed the same trend as bacteria. For fungi, richness in Crops/weeds was significantly higher than Moorland grass-mosaic ($p = 0.002$), Heath/bog, and Lowland as well as Upland wood ($p < 0.001$). Heath/bog ($p < 0.001$), Moorland grass-mosaic ($p = 0.01$), Lowland ($p = 0.006$) and Upland wood ($p < 0.001$) all had significantly lower richness values. Richness of Fertile grassland sites was also higher than all other AVCs ($p < 0.001$) except Crops/weeds. For richness of protists (Fig. 3c), again, the productive Crops/weeds and grassland sites had significantly greater richness than the woodland and upland sites (all $p < 0.001$). Protist richness of Fertile and Infertile grasslands and Lowland wood, Upland wood, Moorland grass-mosaic, were all significantly greater than in Heath/bog as well (all $p < 0.001$).

Supplementary Methods

Sankey diagrams were produced in R[8] using the riverplot package[9]. In brief, proportional abundances at the class-level were calculated on rarefied OTU tables of each organismal group (i.e. bacteria, archaea, etc.) using package phyloseq[10]. Proportions of each class were then assigned to a data frame of “edges”. The data are treated such that the value denotes the distance between “node 1” (i.e. bacteria) and “node 2” (i.e. Proteobacteria .etc.). The names of these nodes are extracted into a new data frame in, which the horizontal and vertical locations of the nodes are determined. Colour was also assigned within this data frame. Finally, the nodes and edges are coerced into a list and converted to an “rp” class object and then presented with the plot function.

Supplementary Table 1. Description of Aggregate Vegetation Classes identified in this study. Adapted from Smart et al.[11].

Aggregate Vegetation Class	Description
Crops/weeds (n = 9)	Communities on disturbed or cultivated land, including weedy, horticultural, and species-poor arable land.
Fertile grassland (n = 98)	Improved or semi-improved grassland. Usually with high nutrient inputs and cut more than once a year.
Infertile Grassland (n = 162)	Semi-improved to unimproved, less productive grasslands, species-rich grasslands including wet or dry and acidic to basic variations.
Lowland wood (n = 17)	Dominated by trees and shrubs in neutral or basic lowlands, scrublands, and hedgerows.
Upland wood (n = 44)	Commonly acidic conifer plantations, scrubland and semi-natural broadleaved woods in the uplands.
Moorland grass/mosaic (n = 54)	Grass-dominated upland pasture, commonly with a long history of livestock grazing.
Heath/bog (n = 52)	Heather dominated, commonly upland landscapes, including dry heath and bogs.

Supplementary Table 2. Pearson's correlation coefficients of the relationship between richness of major groups of soil biota. *** indicates P < 0.001, ** 0.001 > P < 0.01, * 0.01 > P < 0.05, blank indicates P > 0.05.

Taxon	Bacteria	Archaea	Fungi	Protists
**Bacteria**				
**Archaea**	-0.33***			
**Fungi**	0.65***	-0.29***		
**Protists**	0.82***	-0.38***	0.65***	
**Animals**	0.20***	0.04	0.07	0.20***

Supplementary Table 4. Summary of relationships amongst environmental factors and fungal communities derived from NMDS ordination and linear fitting with the envfit function. +/- signify the direction of association between each variable and respective NMDS axes. *** indicates P < 0.001, ** 0.001 > P < 0.01, * 0.01 > P < 0.05, blank indicates P > 0.05.

Variable		Correlation	
	R^2	Axis1	Axis2
pH ($CaCl_2$)	0.6***	-	+
C:N ratio^S	0.47***	+	-
Elevation (m)	0.41***	+	-
Volumetric water content (m^3 m^3 ^-1)	0.41***	+	-
Mean annual precipitation (mL)	0.39***	+	-
Bulk density (g cm^3 ^-1)	0.38***	-	+
Organic matter (% LOI)^L	0.37***	+	-
Total C (%)^L	0.31***	+	-
Clay content (%)^A	0.28***	-	+
Soil bound water (g water g dry soil ^-1)	0.26***	+	-
Soil water repellency^{L*}	0.24***	+	-
Total N (%)^L	0.21***	+	-
Sand content (%)^A	0.19***	+	+
Collembola^{L1}	0.15***	-	+
Total mesofauna^{L1}	0.12***	+	+
Total P (mg kg ^-1)^S	0.11***	-	-
Mites^{L1}	0.1***	+	+
Rock volume (mL)	0.07***	-	+
Temperature (°C)	0.04***	-	+

Note: ^{A} denotes Aitchison's log-ratio transformation; ^{L} denotes log_{10}-transformation; ^{L1} denotes log_{10} plus 1 transformation ^{S} denotes square-root-transformation: * soil water repellency was derived from median water drop penetration times (s).

Supplementary Table 5. Summary of relationships amongst environmental factors and protistan communities derived from NMDS ordination and linear fitting with the envfit function. +/- signify the direction of association between each variable and respective NMDS axes. *** indicates P < 0.001, ** 0.001 > P < 0.01, * 0.01 > P < 0.05, blank indicates P > 0.05.

Variable		Correlation	
	R^2	Axis1	Axis2
pH ($CaCl_2$)	0.6***	-	-
C:N ratio[S]	0.45***	+	-
Elevation (m)	0.43***	+	-
Mean annual precipitation (mL)	0.42***	+	-
Total C (%)[L]	0.4***	+	-
Organic matter (% LOI)[L]	0.39***	+	-
Bulk density (g cm^3 ^-1)	0.37***	-	+
Volumetric water content (m^3 m^3 ^-1)	0.37***	+	-
Clay content (%)[A]	0.28***	-	+
Total N (%)[L]	0.26***	+	-
Soil water repellency[L*]	0.22***	+	-
Soil bound water (g water g dry soil ^-1)	0.2***	+	-
Sand content (%)[A]	0.14***	+	+
Collembola[L1]	0.12***	-	+
Total mesofauna[L1]	0.09***	+	+
Mites[L1]	0.07***	+	+
Total P (mg kg ^-1)[S]	0.07***	-	-
Rock volume (mL)	0.06**	-	+
Temperature (°C)	0.03*	+	+

Note: [A] denotes Aitchison's log-ratio transformation; [L] denotes log_{10}-transformation; [L1] denotes log_{10} plus 1 transformation [S] denotes square-root-transformation; * soil water repellency was derived from median water drop penetration times (s).

Supplementary Table 3. Summary of relationships amongst environmental factors and archaea communities derived from NMDS ordination and linear fitting with the envfit function. +/- signify the direction of association between each variable and respective NMDS axes. *** indicates P < 0.001, ** 0.001 > P < 0.01, * 0.01 > P < 0.05, blank indicates P > 0.05.

Variable		Correlation	
	R^2	Axis1	Axis2
pH ($CaCl_2$)	0.57***	+	-
C:N ratio[S]	0.49***	-	+
Elevation (m)	0.48***	-	+
Bulk density (g cm^3 ^-1)	0.41***	+	+
Mean annual precipitation (mL)	0.35***	-	-
Organic matter (% LOI)[L]	0.34***	-	-
Total C (%)[L]	0.34***	-	-
Clay content (%)[A]	0.31***	+	+
Volumetric water content (m^3 m^3 ^-1)	0.3***	-	-
Soil bound water (g water g dry soil ^-1)	0.24***	-	-
Soil water repellency[L*]	0.24***	-	+
Total N (%)[L]	0.17***	-	-
Total P (mg kg ^-1)[S]	0.12***	+	-
Sand content (%)[A]	0.1***	-	+
Collembola[L1]	0.06***	+	+
Total mesofauna[L1]	0.05**	-	+
Mites[L1]	0.05**	-	+
Temperature (°C)	0.05**	-	+
Rock volume (mL)	0.04*	+	+

Note: [A] denotes Aitchison's log-ratio transformation; [L] denotes log_{10}-transformation; [L1] denotes log_{10} plus 1 transformation [S] denotes square-root-transformation; * soil water repellency was derived from median water drop penetration times (s).

Supplementary Table 6. Summary of relationships amongst environmental factors and animal communities derived from NMDS ordination and linear fitting with the envfit function. +/- signify the direction of association between each variable and respective NMDS axes. *** indicates $P < 0.001$, ** $0.001 > P < 0.01$, * $0.01 > P < 0.05$, blank indicates $P > 0.05$.

Variable		Correlation	
	R^2	Axis1	Axis2
pH ($CaCl_2$)	0.48***	-	+
Bulk density (g cm^3 ^-1)	0.43***	-	-
C:N ratio^S	0.35***	+	+
Organic matter (% LOI)^L	0.35***	+	+
Volumetric water content (m^3 m^3 ^-1)	0.32***	+	+
Total C (%)^L	0.29***	+	+
Elevation (m)	0.28***	+	+
Soil water repellency^{L*}	0.27***	+	-
Mean annual precipitation (mL)	0.24***	+	+
Clay content (%)^A	0.22***	-	-
Total N (%)^L	0.2***	+	+
Soil bound water (g water g dry soil^-1)	0.2***	+	+
Mites^{L1}	0.11***	+	-
Total mesofauna^{L1}	0.1***	+	-
Sand content (%)^A	0.08***	+	-
Rock volume (mL)	0.08***	-	-
Total P (mg kg ^-1)^S	0.06***	-	+
Collembola^{L1}	0.05**	-	-
Temperature (°C)	0.03*	+	-

Note: ^{A} denotes Aitchison's log-ratio transformation; ^{L} denotes log_{10}-transformation; ^{L1} denotes log_{10} plus 1 transformation ^{S} denotes square-root-transformation: * soil water repellency was derived from median water drop penetration times (s).

Supplementary Table 7. Summary of relationships amongst environmental factors and bacterial communities derived from CAP ordination and linear fitting with the envfit function. *** indicates P < 0.001, ** 0.001 > P < 0.01, * 0.01 > P < 0.05, blank indicates P > 0.05.

Soil and environmental variables	R^2
pH ($CaCl_2$)	0.66***
Mean annual precipitation (mL)	0.51***
C:N ratio^S	0.48***
Elevation (m)	0.47***
Volumetric water content (m^3 m^3 ^-1)	0.46***
Bulk density (g cm^3 ^-1)	0.44***
Organic matter (% LOI)^L	0.39***
Total C (%)^L	0.32***
Clay content (%)^A	0.29***
Soil bound water (g water g dry soil ^-1)	0.26***
Soil water repellency^L	0.26***
Sand content (%)^A	0.22***
Total N (%)^L	0.22***
Total P (mg kg ^-1)^S	0.09***
Collembola^{L1}	0.09***
Total mesofauna^{L1}	0.08***
Mites^{L1}	0.08***
Rock volume (mL)	0.05***
Temperature (°C)	0.04**

Note: ^{A} denotes Aitchison's log-ratio transformation; ^{L} denotes log_{10}-transformation; ^{L1} denotes log_{10} plus 1 transformation ^{S} denotes square-root-transformation: * soil water repellency was derived from median water drop penetration times (s).

Supplementary Table 8. Summary of relationships amongst environmental factors and fungal communities derived from CAP ordination and linear fitting with the envfit function. *** indicates $P < 0.001$, ** $0.001 > P < 0.01$, * $0.01 > P < 0.05$, blank indicates $P > 0.05$.

Soil and environmental variables	R^2
C:N ratio^S	0.43***
Elevation (m)	0.35***
pH ($CaCl_2$)	0.35***
Volumetric water content (m^3 m^3 ^-1)	0.34***
Bulk density (g cm^3 ^-1)	0.30***
Mean annual precipitation (mL)	0.22***
Sand content (%)^A	0.20***
Organic matter (% LOI)^L	0.20***
Clay content (%)^A	0.20***
Total C (%)^L	0.18***
Soil water repellency^L	0.18***
Soil bound water (g water g dry soil ^-1)	0.14***
Collembola^{L1}	0.09***
Total P (mg kg ^-1)^S	0.09***
Total mesofauna^{L1}	0.07***
Mites^{L1}	0.07***
Total N (%)^L	0.07***
Rock volume (mL)	0.06**
Temperature (°C)	0.06***

Note: ^{A} denotes Aitchison's log-ratio transformation; ^{L} denotes log_{10}-transformation; ^{L1} denotes log_{10} plus 1 transformation ^{S} denotes square-root-transformation: * soil water repellency was derived from median water drop penetration times (s).

Supplementary Table 9. Summary of relationships amongst environmental factors and protistan communities derived from CAP ordination and linear fitting with the envfit function. *** indicates P < 0.001, ** 0.001 > P < 0.01, * 0.01 > P < 0.05, blank indicates P > 0.05.

Soil and environmental variables	R^2
pH ($CaCl_2$)	0.59***
C:N ratio^S	0.46***
Total C (%)^L	0.41***
Organic matter (% LOI)^L	0.40***
Bulk density (g cm^3 ^-1)	0.40***
Elevation (m)	0.38***
Mean annual precipitation (mL)	0.33***
Clay content (%)^A	0.27***
Volumetric water content (m^3 m^3 ^-1)	0.27***
Total N (%)^L	0.26***
Soil water repellency^L	0.24***
Soil bound water (g water g dry soil ^-1)	0.24***
Sand content (%)^A	0.24***
Total P (mg kg ^-1)^S	0.15***
Total mesofauna^{L1}	0.10***
Collembola^{L1}	0.10***
Mites^{L1}	0.09***
Rock volume (mL)	0.03*
Temperature (°C)	0.03*

Note: ^{A} denotes Aitchison's log-ratio transformation; ^{L} denotes log_{10}-transformation; ^{L1} denotes log_{10} plus 1 transformation ^{S} denotes square-root-transformation: * soil water repellency was derived from median water drop penetration times (s).

Supplementary Table 10. Summary of relationships amongst environmental factors and archaeal communities derived from CAP ordination and linear fitting with the envfit function. *** indicates P < 0.001, ** 0.001 > P < 0.01, * 0.01 > P < 0.05, blank indicates P > 0.05.

Soil and environmental variables	R^2
pH ($CaCl_2$)	0.60***
Elevation (m)	0.45***
Bulk density (g cm^3 ^-1)	0.44***
C:N ratio[S]	0.42***
Total C (%)[L]	0.35***
Organic matter (% LOI)[L]	0.35***
Mean annual precipitation (mL)	0.33***
Clay content (%)[A]	0.30***
Volumetric water content (m^3 m^3 ^-1)	0.28***
Soil bound water (g water g dry soil ^-1)	0.27***
Soil water repellency[L]	0.24***
Total N (%)[L]	0.21***
Total P (mg kg ^-1)[S]	0.10***
Sand content (%)[A]	0.06**
Collembola[L1]	0.06***
Mites[L1]	0.06**
Total mesofauna[L1]	0.05**
Temperature (°C)	0.05**
Rock volume (mL)	0.02

Note: [A] denotes Aitchison's log-ratio transformation; [L] denotes log_{10}-transformation; [L1] denotes log_{10} plus 1 transformation [S] denotes square-root-transformation: * soil water repellency was derived from median water drop penetration times (s).

Supplementary Table 11. Summary of relationships amongst environmental factors and animal communities derived from CAP ordination and linear fitting with the envfit function. *** indicates $P < 0.001$, ** $0.001 > P < 0.01$, * $0.01 > P < 0.05$, blank indicates $P > 0.05$.

Soil and environmental variables	R^2
pH ($CaCl_2$)	0.47***
Volumetric water content (m^3 m^3 ^-1)	0.35***
C:N ratio^S	0.29***
Bulk density (g cm^3 ^-1)	0.26***
Elevation (m)	0.26***
Organic matter (% LOI)^L	0.25***
Total C (%)^L	0.21***
Soil water repellency^L	0.20***
Clay content (%)^A	0.20***
Mean annual precipitation (mL)	0.19***
Total N (%)^L	0.14***
Sand content (%)^A	0.13***
Soil bound water (g water g dry soil ^-1)	0.13***
Total mesofauna^{L1}	0.11***
Mites^{L1}	0.10***
Collembola^{L1}	0.08***
Total P (mg kg ^-1)^S	0.08***
Temperature (°C)	0.07***
Rock volume (mL)	0.06***

Note: ^{A} denotes Aitchison's log-ratio transformation; ^{L} denotes $\log_{10}$-transformation; ^{L1} denotes $\log_{10}$ plus 1 transformation ^{S} denotes square-root-transformation: * soil water repellency was derived from median water drop penetration times (s).

Supplementary Table 12. Mean values (± SE) of soil physical and chemical variables of each Aggregate Vegetation Class (AVC). Following normalisation on selected variables (see notes of Supplementary Tables 2-5) ANOVAs and Tukey's *post-hoc* tests were performed. Results are as follows: total C ($F_{6, 427} = 89.13$ $p < 0.001$), total N ($F_{6, 427} = 61.03$, $p < 0.001$), C :N ratio ($F_{6, 427} = 94.41$, $p < 0.001$), total P ($F_{6, 424} = 7.1$, $p < 0.001$), organic matter ($F_{6, 428} = 107.02$, $p < 0.001$), pH ($F_{6, 428} = 69.56$, $p < 0.001$), soil water repellency ($F_{6, 428} = 22.08$, $p < 0.001$), volumetric water content ($F_{6, 427} = 33.74$, $p < 0.001$), soil bound water ($F_{6, 428} = 79.87$, $p < 0.001$), rock volume ($F_{6, 427} = 10.4$, $p < 0.001$), bulk density ($F_{6, 427} = 90.99$, $p < 0.001$), clay content ($F_{6, 344} = 19.54$, $p < 0.001$), sand content ($F_{6, 344} = 5.71$, $p < 0.001$), elevation ($F_{6, 429} = 78.42$, $p < 0.001$), mean annual precipitation ($F_{6, 429} = 72.6$, $p < 0.001$), and temperature ($F_{6, 429} = 4.4$, $p < 0.001$).

Environmental variable	Crops/weeds	Fertile grassland	Infertile grassland	Lowland wood	Upland wood	Moorland grass-mosaic	Heath/bog
otal C (%)	3.87 (± 0.83)d	4.75 (± 0.2)d	5.85 (± 0.33)d	5.78 (± 1.07)d	9.7 (± 2.25)c	12.19 (± 2.07)b	23.57 (± 1.88)a
otal N (%)	0.32 (± 0.05)d	0.45 (± 0.02)d	0.49 (± 0.02)d	0.4 (± 0.06)d	0.58 (± 0.1)c	0.83 (± 0.11)b	1.05 (± 0.09)a
:N ratio	11.44 (± 0.81)cd	10.49 (± 0.13)d	11.62 (± 0.27)cd	13.92 (± 0.75)bc	15.86 (± 0.7)b	14.41 (± 0.42)b	20.65 (± 0.94)a
otal P (mg kg^-1)	1103.44 (± 145.47)ab	1194.9 (± 45.53)a	1045.5 (± 43.3)ab	601.68 (± 77.68)c	762.45 (± 61.95)bc	930.49 (± 57.5)ab	769.63 (± 50.04)ab
rganic matter (% LOI)	7.53 (± 1.62)d	9.39 (± 0.34)d	11.25 (± 0.55)d	10.71 (± 1.7)d	18.79 (± 4.16)c	22.99 (± 3.72)b	39.26 (± 3.6)a
H ($CaCl_2$)	4.73 (± 0.26)b	5.2 (± 0.08)a	4.73 (± 0.05)b	4.31 (± 0.26)b	3.57 (± 0.1)cd	3.85 (± 0.09)c	3.84 (± 0.1)d
oil water repellency*	4077.56 (± 3990.72)abc	264.01 (± 73.28)c	781.68 (± 137.58)b	2975.47 (± 2108.12)abc	1965.87 (± 698.61)a	4186.13 (± 798.48)a	3186.4 (± 812.15)a
olumetric water content m^3 m^3 ^-1)	0.23 (± 0.03)bc	0.35 (± 0.01)b	0.34 (± 0.01)b	0.22 (± 0.02)c	0.36 (± 0.03)b	0.46 (± 0.02)a	0.52 (± 0.02)a
oil bound water (g ater g dry soil ^-1)	2.19 (± 0.32)c	2.74 (± 0.11)c	2.89 (± 0.11)c	2.92 (± 0.34)c	3.7 (± 0.49)b	4.45 (± 0.46)b	6.03 (± 0.47)a
ock volume (mL)	3.95 (± 1.11)abc	5.25 (± 0.45)b	5.44 (± 0.42)b	9.13 (± 2.49)a	4.41 (± 0.57)ab	3.25 (± 0.39)c	1.87 (± 0.21)c
ulk density (g cm^3 ^-1)	1.03 (± 0.09)a	0.9 (± 0.02)a	0.8 (± 0.02)b	0.71 (± 0.08)b	0.56 (± 0.04)c	0.5 (± 0.04)c	0.47 (± 0.03)d
lay content (%)	22.25 (± 1.85)ab	25.46 (± 0.65)a	23.18 (± 0.64)ab	17.47 (± 1.34)ab	17.82 (± 1.82)ab	18.12 (± 1.27)c	11.76 (± 2.24)d
and content (%)	30.97 (± 4.66)ad	24.88 (± 1.25)d	29.21 (± 1.44)bd	42.99 (± 4.01)ac	40.23 (± 4.15)abc	29.5 (± 3.0)b	45.15 (± 7.61)a
levation (m)	88.71 (± 47.69)cd	109.38 (± 8.62)d	167.28 (± 8.65)c	119.06 (± 16.38)cd	297.83 (± 20.62)b	406.63 (± 19.22)a	380.55 (± 19.7)a
ean annual recipitation (mL)	968.44 (± 69.01)c	1078.19 (± 24.71)c	1177.05 (± 18.91)c	1100.12 (± 52.28)c	1405.33 (± 65.35)b	2027.23 (± 74.39)a	1771.2 (± 58.19)a
emperature (°C)	12.64 (± 1.18)ab	12.09 (± 0.41)b	13.44 (± 0.29)a	15.8 (± 0.87)a	14.53 (± 0.53)a	14.51 (± 0.36)a	13.87 (± 0.29)a

*Soil water repellency was derived from median water drop penetration times (s) and log transformed.

Supplementary Table 13. UK soil groups listed with their complementary classification in the FAO World Reference Base Classification[12]. Soils are listed in alphabetical order.

Major UK soil group	World Reference Base
Brown	Primarily Cambisols plus some Luvisols and Acrisols
Lithomorphic	Leptosols with some Regosols
Surface- and ground-water gleys	Primarily Gleysols, Planosols, and some Fluvisols/Luvisols
Podzolic	Podzols
Peat	Histosols
Man made	Anthrosols

Supplementary Table 14. Rarefaction depth and a breakdown of replicate numbers for each taxonomic group.

Taxon	Rarefaction depth (reads)	Replicates per Aggregate Vegetation Class
**Bacteria**	40,000	Crops/weeds = 9<br>Fertile grassland = 96<br>Infertile grassland = 157<br>Lowland wood = 17<br>Upland wood = 43<br>Moorland grass-mosaic = 54<br>Heath/bog = 52
**Archaea**	200	Crops/weeds = 9<br>Fertile grassland = 87<br>Infertile grassland = 91<br>Lowland wood = 15<br>Upland wood = 42<br>Moorland grass-mosaic = 48<br>Heath/bog = 51
**Fungi**	4,000	Crops/weeds = 9<br>Fertile grassland = 97<br>Infertile grassland = 156<br>Lowland wood = 17<br>Upland wood = 43<br>Moorland grass-mosaic = 44<br>Heath/bog = 47
**Protists**	15,000	Crops/weeds = 9<br>Fertile grassland = 98<br>Infertile grassland = 160<br>Lowland wood = 17<br>Upland wood = 40<br>Moorland grass-mosaic = 46<br>Heath/bog = 42
**Animals**	1,000	Crops/weeds = 7<br>Fertile grassland = 91<br>Infertile grassland = 144<br>Lowland wood = 17<br>Upland wood = 44<br>Moorland grass-mosaic = 53<br>Heath/bog = 52

Supplementary Figures

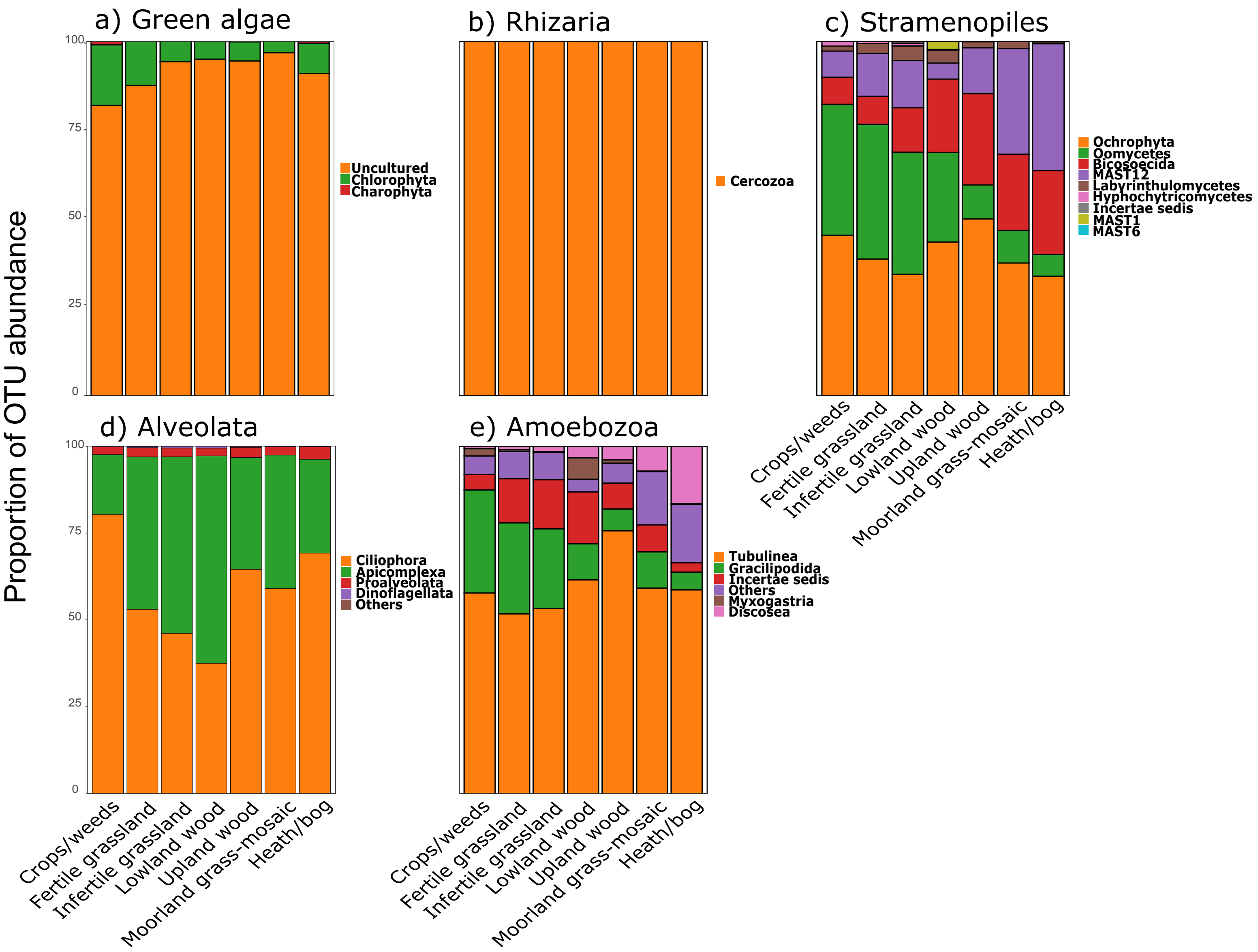

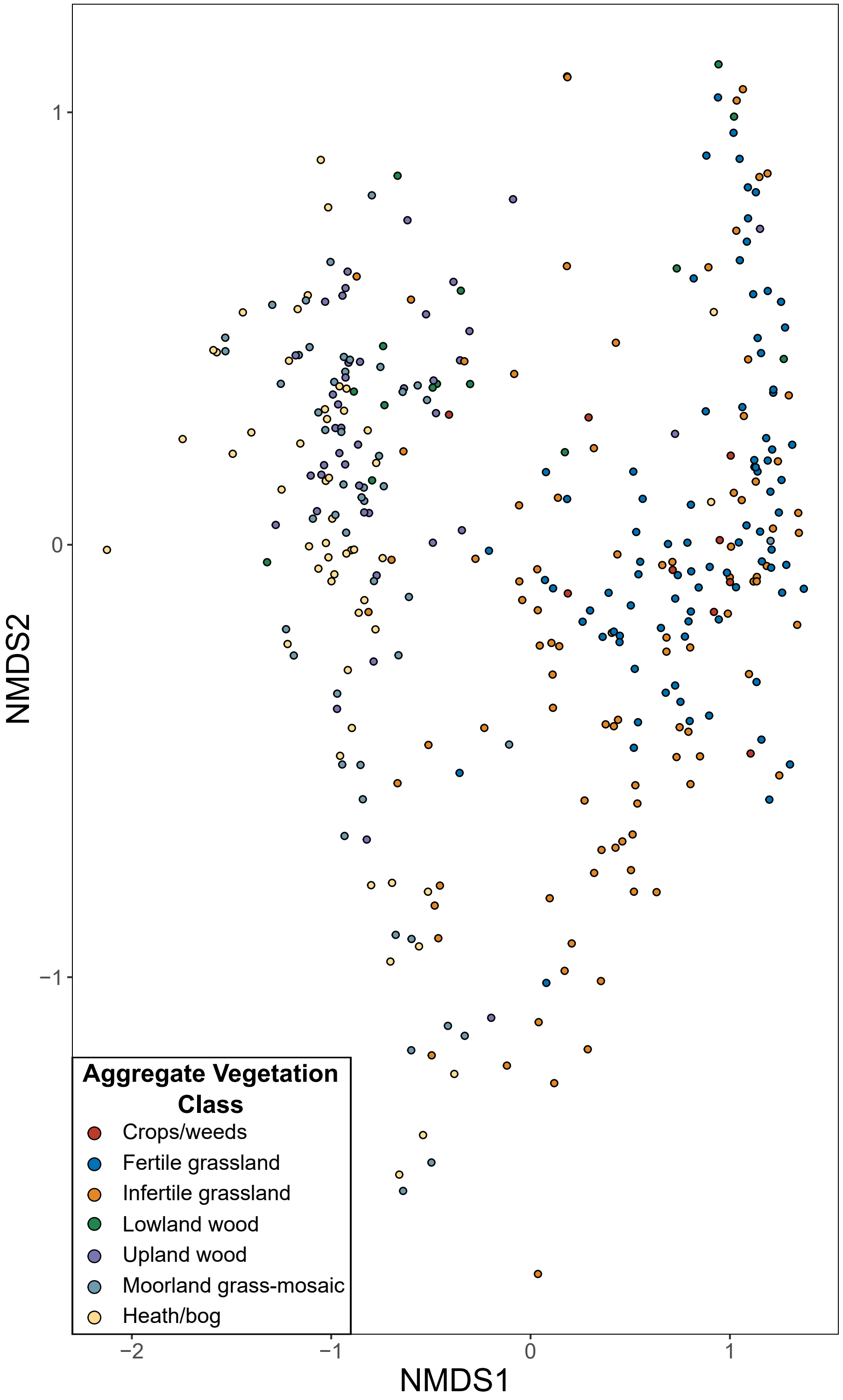

Supplementary Fig. 2. Plot of the non-metric dimensional scaling ordination (stress = 0.11) of archaea community composition across GMEP sites. Samples are coloured by Aggregate Vegetation Class. Results of PERMANOVA ($F_{6,336} = 15.32$, p = 0.001) and of dispersion of variances ($F_{6,336} = 8.52$, p = 0.001) were significant.

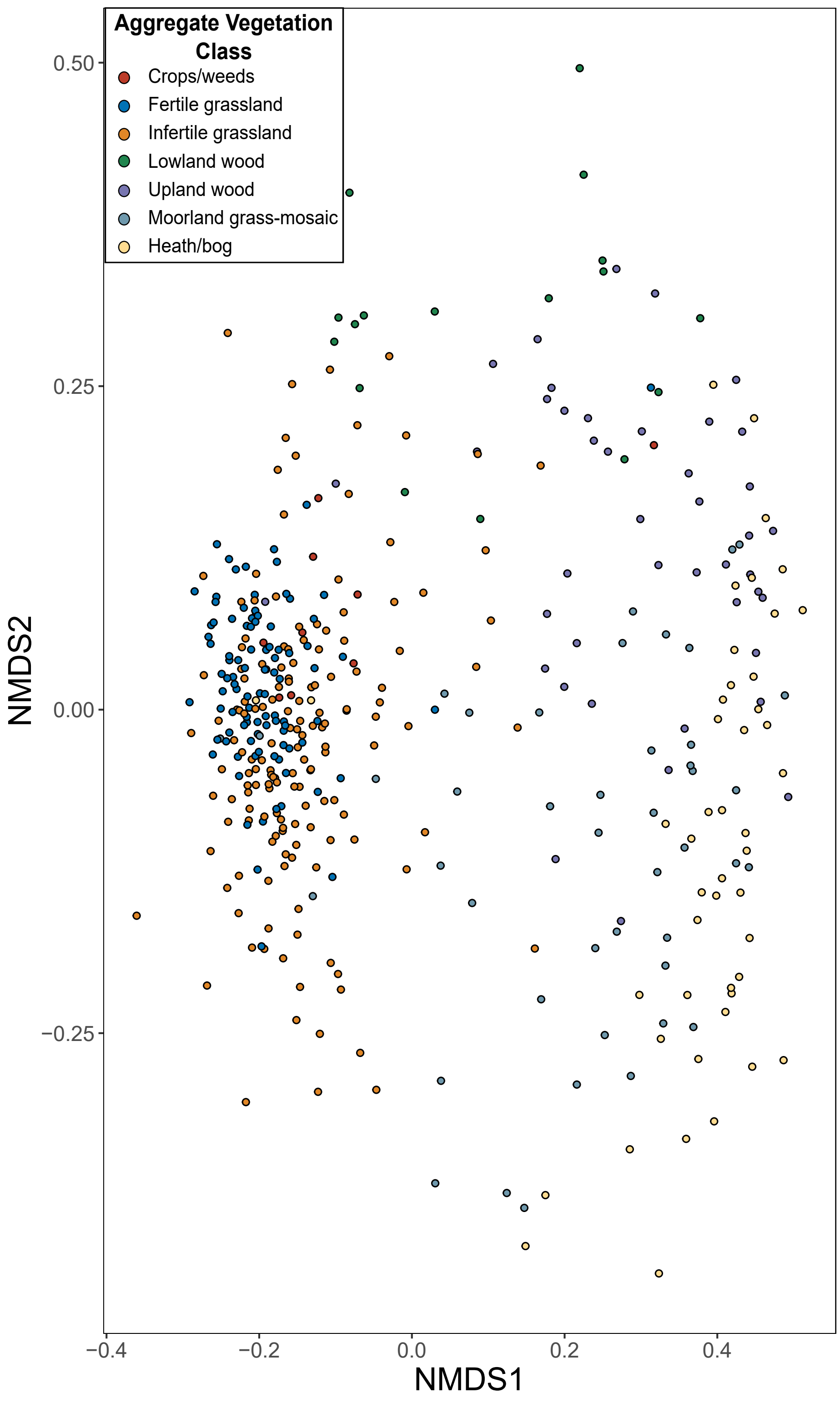

Supplementary Fig. 3 Plot of the non-metric dimensional scaling ordination (stress = 0.13) of fungi community composition across GMEP sites. Samples are coloured by Aggregate Vegetation Class. Results of PERMANOVA ($F_{6,406} = 10.74$, $p = 0.001$) and of dispersion of variances ($F_{6,406} = 41.30$, $p = 0.001$) were significant.

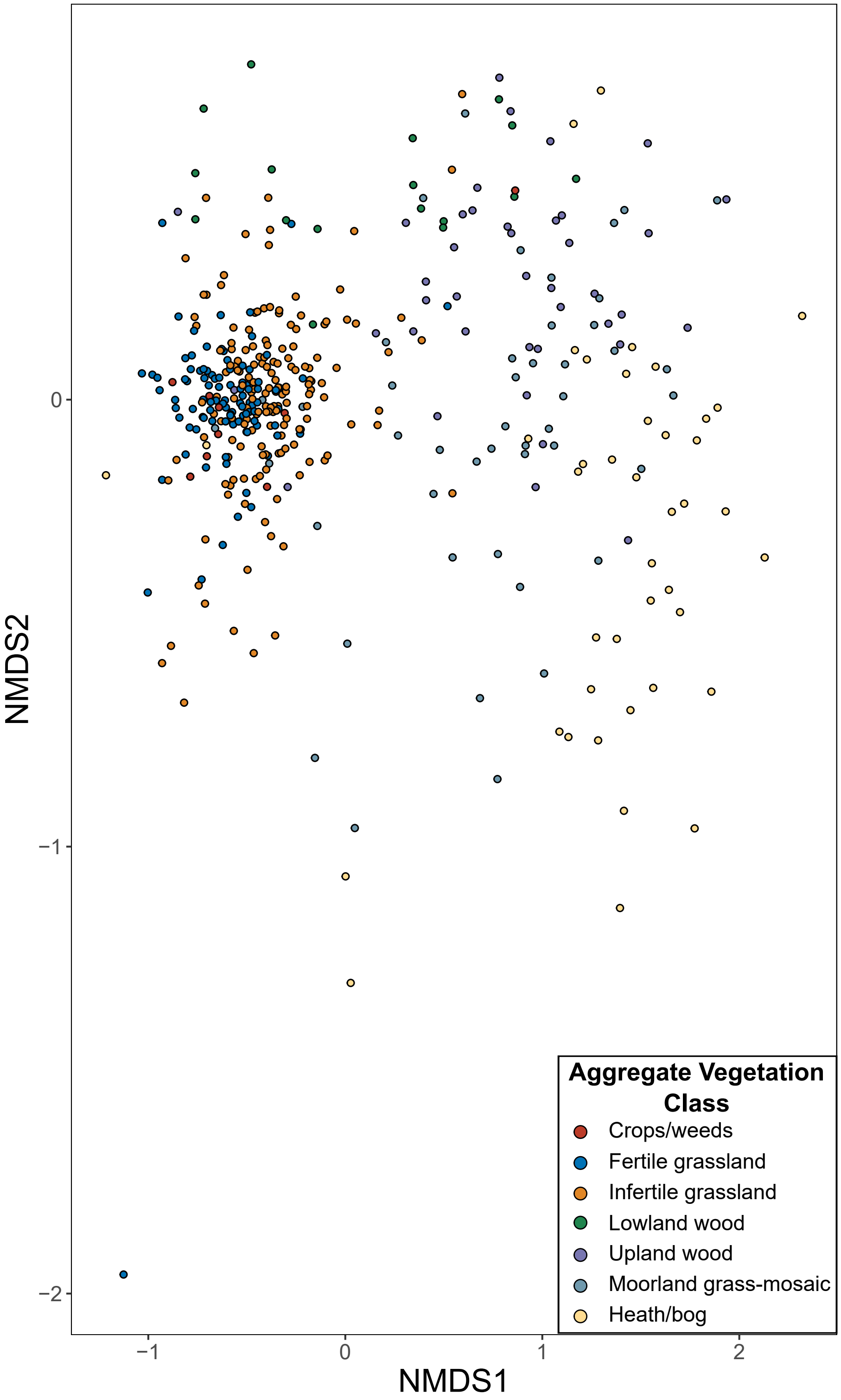

Supplementary Fig. 4. Plot of the non-metric dimensional scaling ordination (stress = 0.08) of protist community composition across GMEP sites. Samples are coloured by Aggregate Vegetation Class. Results of PERMANOVA ($F_{6,405}$= 31.60, p = 0.001) and of dispersion of variances ($F_{6,405}$ = 17.63, p = 0.001) were significant.

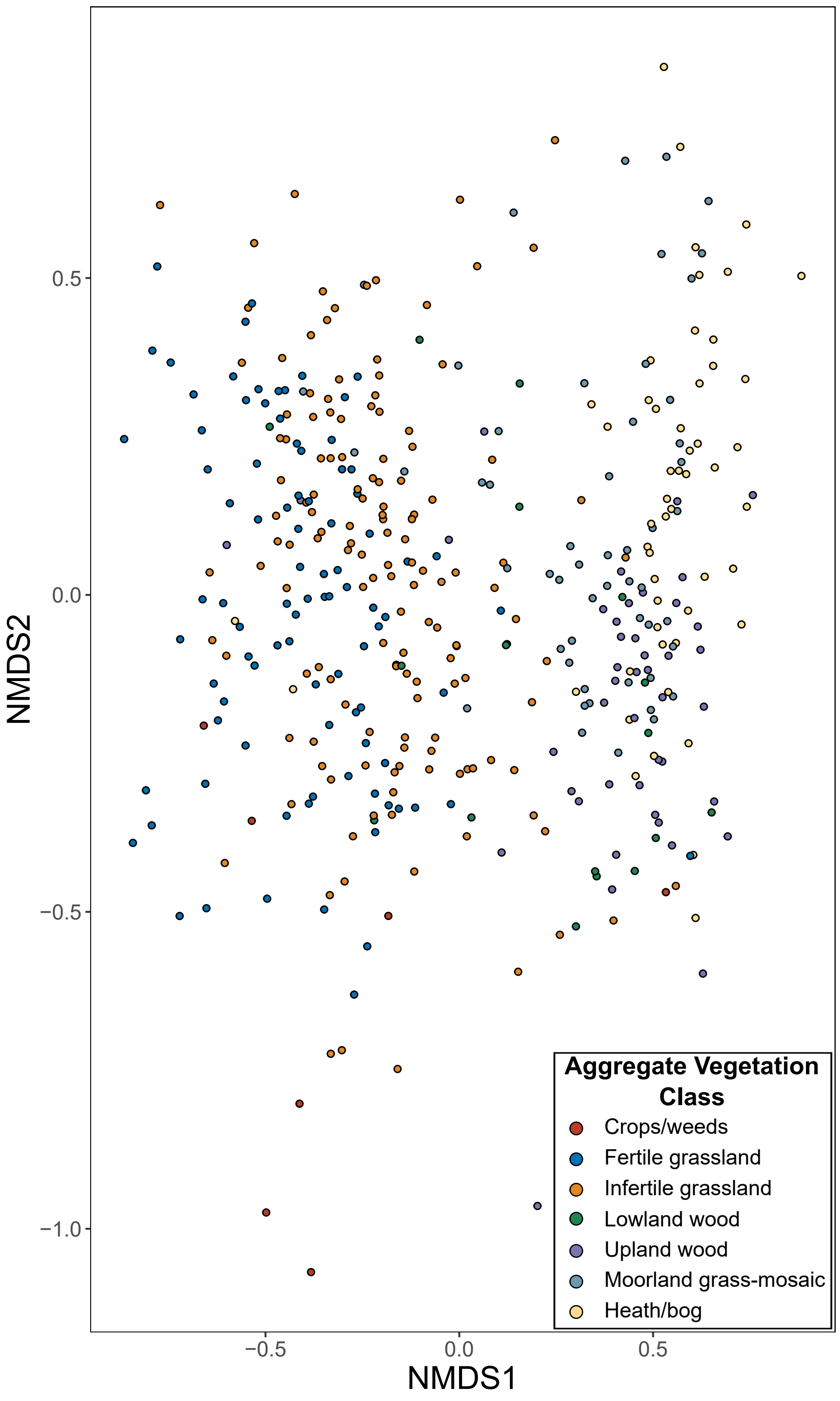

Supplementary Fig. 5. Plot of the non-metric dimensional scaling ordination (stress = 0.19) of animal community composition across GMEP sites. Samples are coloured by Aggregate Vegetation Class. The PERMANOVA was significant ($F_{6,401} = 7.4$, $p = 0.001$) but not significant differences in dispersion of variances ($F_{6,401} = 8.52$, $p = 0.58$) were observed.

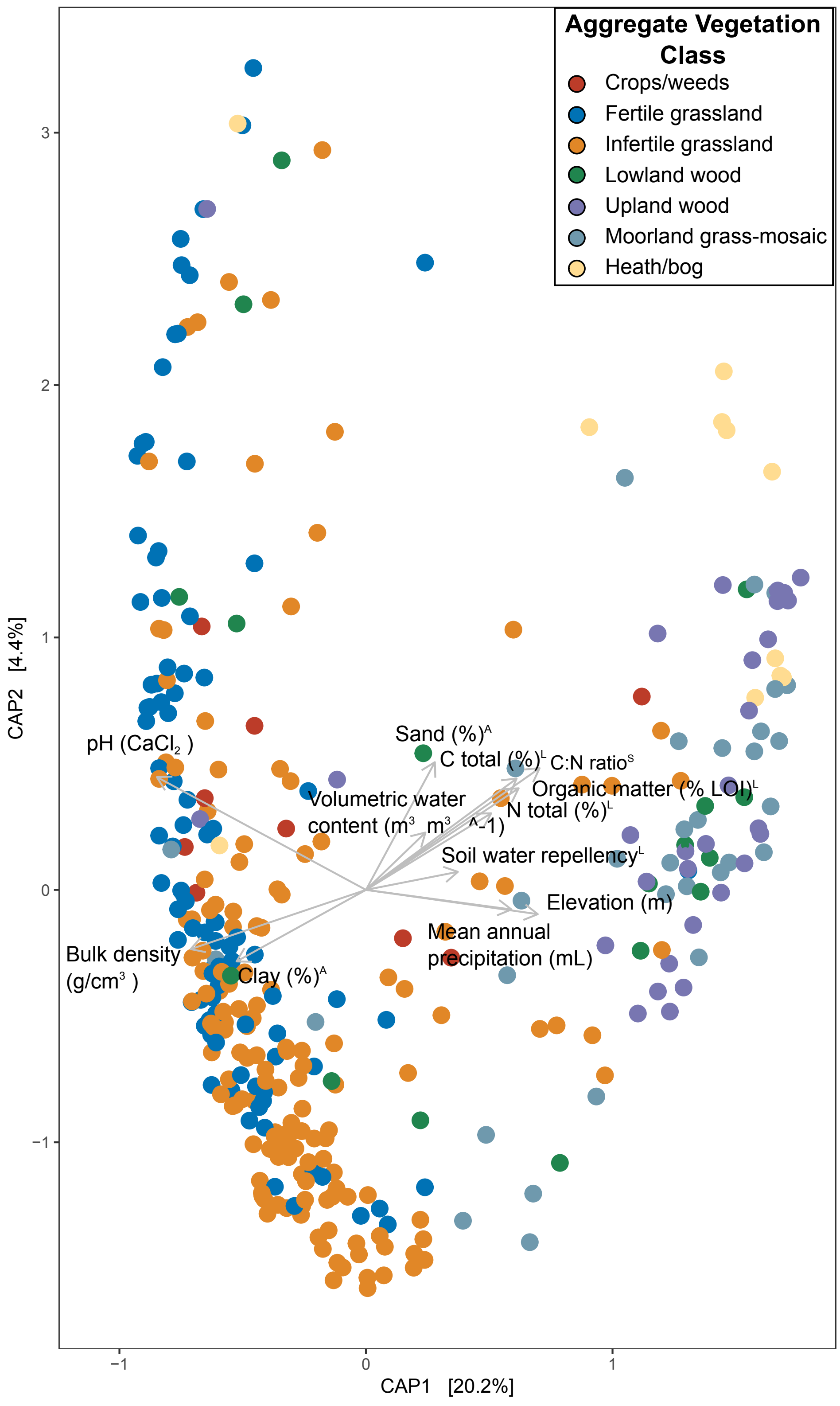

Supplementary Fig. 6. Vector-loading plot of the canonical analysis of principle coordinates constrained ordination of bacterial community composition across GMEP sites. Samples are coloured by Aggregate Vegetation Class. Only variables with $R^2 > 0.2$ from linear fitting were mapped on this ordination.

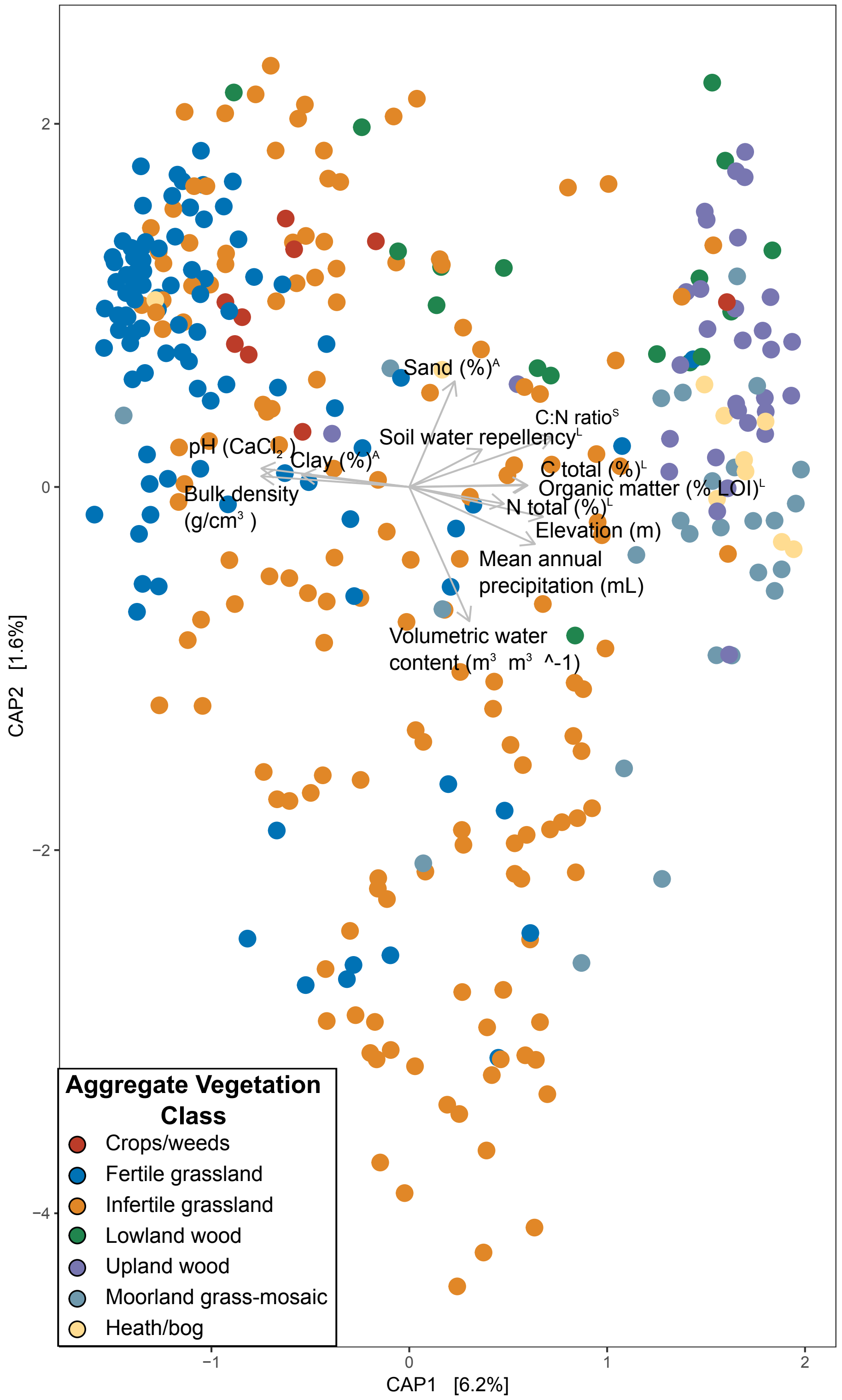

Supplementary Fig. 7. Vector-loading plot of the canonical analysis of principle coordinates constrained ordination of fungal community composition across GMEP sites. Samples are coloured by Aggregate Vegetation Class. Only variables with $R^2 > 0.2$ from linear fitting were mapped on this ordination.

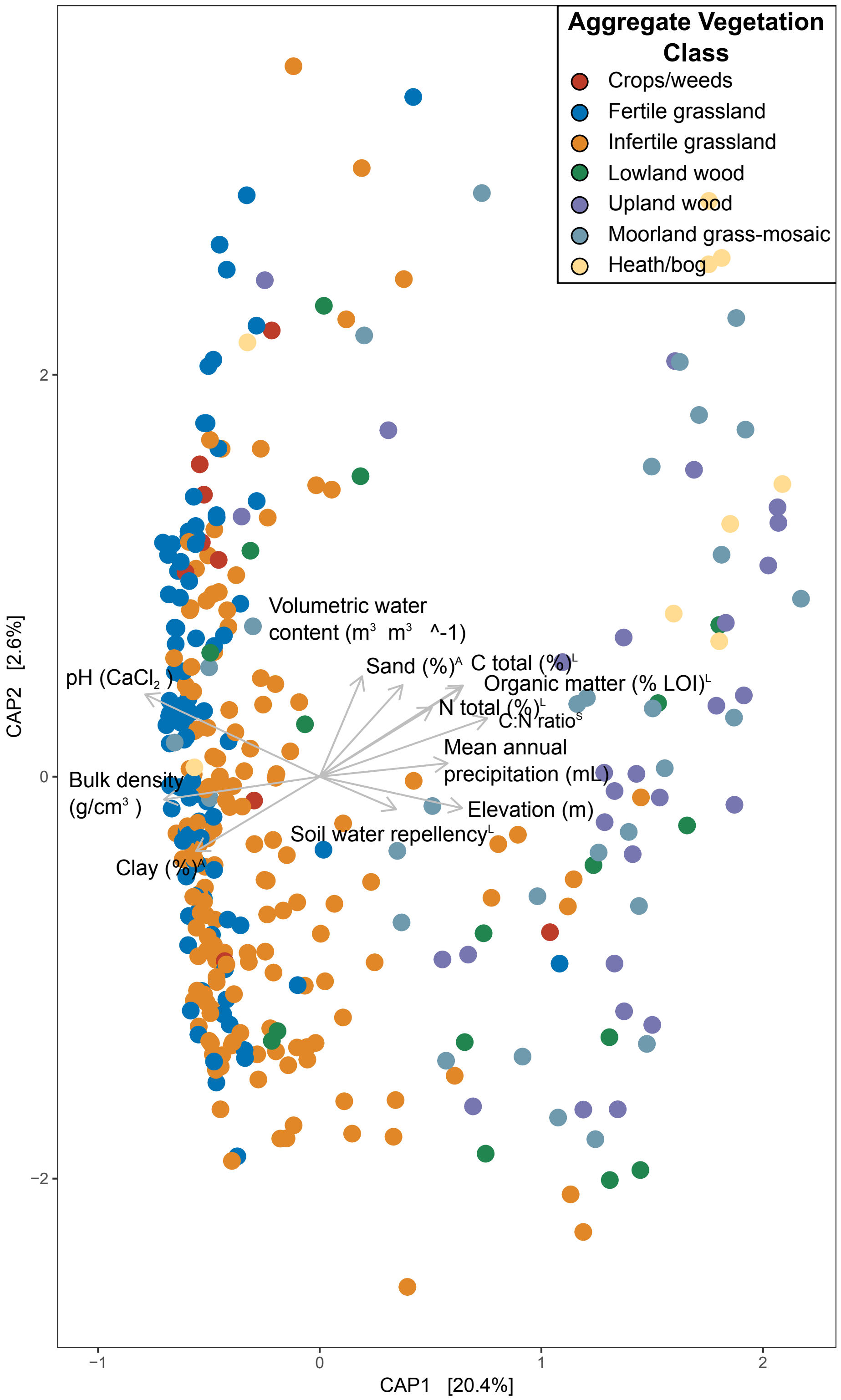

Supplementary Fig. 8. Vector-loading plot of the canonical analysis of principle coordinates constrained ordination of protistan community composition across GMEP sites. Samples are coloured by Aggregate Vegetation Class. Only variables with $R^2 > 0.2$ from linear fitting were mapped on this ordination.

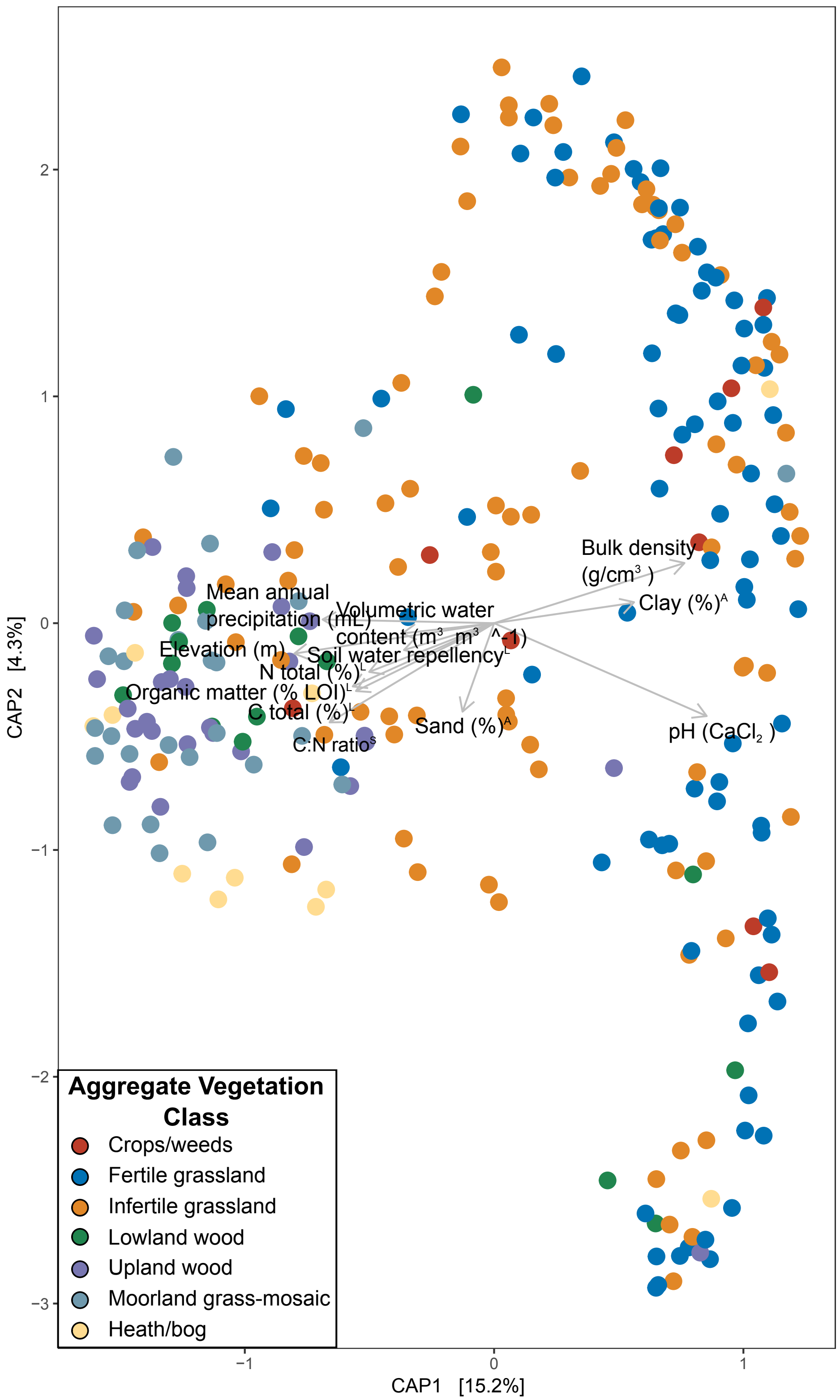

Supplementary Fig. 9. Vector-loading plot of the canonical analysis of principle coordinates constrained ordination of archaeal community composition across GMEP sites. Samples are coloured by Aggregate Vegetation Class. Only variables with $R^2 > 0.2$ from linear fitting were mapped on this ordination.

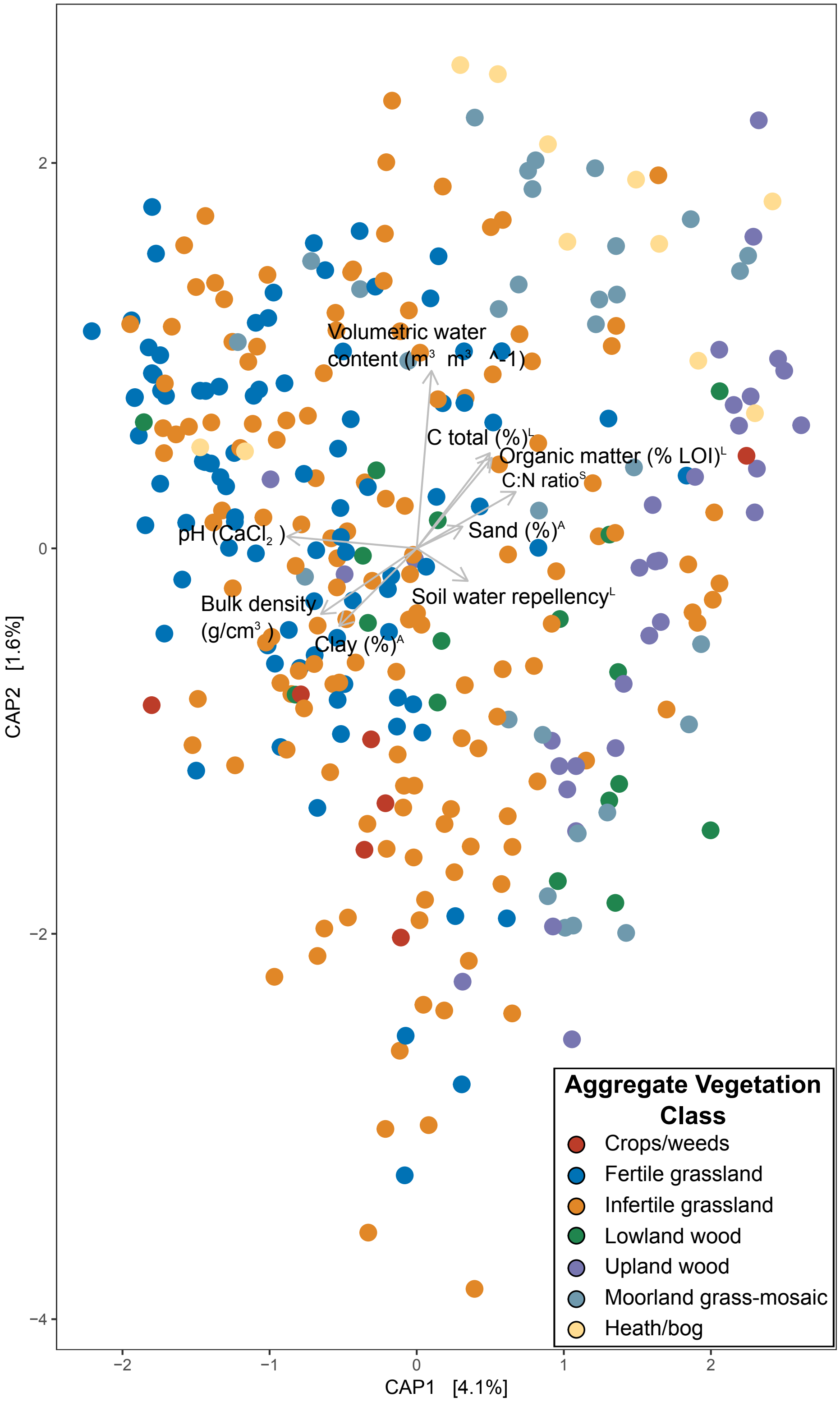

Supplementary Fig. 10. Vector-loading plot of the canonical analysis of principle coordinates constrained ordination of animal community composition across GMEP sites. Samples are coloured by Aggregate Vegetation Class. Only variables with $R^2 > 0.2$ from linear fitting were mapped on this ordination.

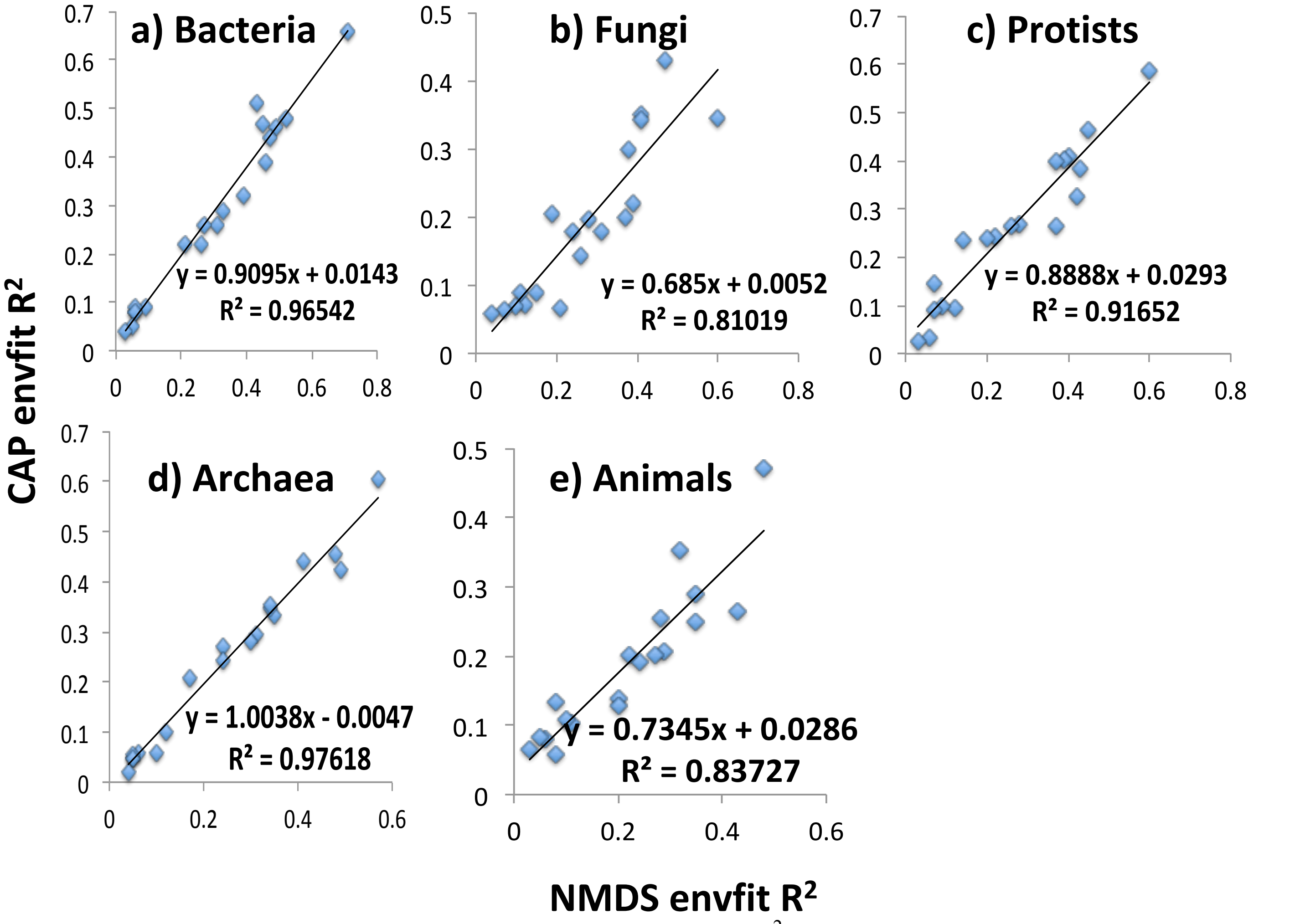

Supplementary Fig. 11. Regressions of goodness-of-fit values (R^2) of environmental variables calculated from linear fitting to NMDS ordinations versus those from CAP ordinations. Equations and R^2 values are shown for **a**) bacteria; **b**) fungi, **c**) protists; **d**) archaea; and **e**) animals.

Supplementary References

1. Hill, M.O. TWINSPAN – a FORTRAN program for arranging multivariate data in an ordered two-way table by classification of the individuals and attributes. (Cornell University, Ithaca, 1979).
2. Hill, M.O. DECORANA – a FORTRAN program for detrended correspondence analysis and reciprocal averaging (Cornell University, Ithaca, 1979).
3. Bunce, R.G.H. et al. Vegetation of the British countryside – the countryside vegetation system. (Department of the Environment, Transport and the Regions, London, 1990).
4. Emmett, B. A. & the GMEP Team. Glastir Monitoring & Evaluation Programme. Final Report to Welsh Government (Contract reference: C147/2010/11. NERC/Centre for Ecology & Hydrology (CEH Project: NEC04780/NEC05371/NEC05782) (2017).
5. Cranfield University. The National Soil Map and Soil Classification. Cranfield Soil and Agrifood Institute, Cranfield University, Bedfordshire, UK (2004).
6. Avery, B.W. Soil classifications for England and Wales (Higher Categories). Soil Survey Technical Monograph No 14. Harpenden, UK (1980).
7. Clayden, B. & Hollis, J.M. Criteria for Differentiating Soil Series. Soil Survey Technical Monograph No 17. Harpenden, UK (1984).
8. R Core Team (2017). R: A language and environment for statistical computing. R Foundation for Statistical Computing, Vienna, Austria (2017). https://www.R-project.org/.
9. Weiner, J. riverplot: Sankey or Ribbon Plots. R package version 0.6 https://CRAN.R-project.org/package=riverplot (2017).
10. McMurdie, P. J. & Holmes, S. phyloseq: an R package for reproducible interactive analysis and graphics of microbiome census data. *PLoS ONE* **8**, e61217 (2013). DOI: 10.1371/journal.pone.0061217
11. Smart, S.M., Robertson, J.C., Shield, E.J., Van de Poll, H.M. Locating eutrophication effects across British vegetation between 1990 and 1998. *Glob. Change Biol.* **9**, 1763-1774 (2003).
12. World Reference Base for Soil Resources. Report 103. Food and Agriculture Organization of the United Nations, Rome (2006).
