# Supplementary material for: Divergent national-scale trends of microbial and animal biodiversity revealed across diverse temperate soil ecosystems
Source: Nat Commun. 2019 Mar 7;10:1107. doi: 10.1038/s41467-019-09031-1 (PMC6405921; doi:10.1038/s41467-019-09031-1)
Supplement: Supplementary file 3 — Reporting Summary [file 41467_2019_9031_MOESM3_ESM.pdf]

nature research

Corresponding author(s): Paul B.L. George NCOMMS-18-31555A

Reporting Summary

Nature Research wishes to improve the reproducibility of the work that we publish. This form provides structure for consistency and transparency in reporting. For further information on Nature Research policies, see Authors & Referees and the Editorial Policy Checklist.

Statistical parameters

When statistical analyses are reported, confirm that the following items are present in the relevant location (e.g. figure legend, table legend, main text, or Methods section).

n/a	Confirmed	
☐	☒	The exact sample size (*n*) for each experimental group/condition, given as a discrete number and unit of measurement
☐	☒	An indication of whether measurements were taken from distinct samples or whether the same sample was measured repeatedly
☐	☒	The statistical test(s) used AND whether they are one- or two-sided<br>*Only common tests should be described solely by name; describe more complex techniques in the Methods section.*
☐	☒	A description of all covariates tested
☐	☒	A description of any assumptions or corrections, such as tests of normality and adjustment for multiple comparisons
☐	☒	A full description of the statistics including central tendency (e.g. means) or other basic estimates (e.g. regression coefficient) AND variation (e.g. standard deviation) or associated estimates of uncertainty (e.g. confidence intervals)
☐	☒	For null hypothesis testing, the test statistic (e.g. *F*, *t*, *r*) with confidence intervals, effect sizes, degrees of freedom and *P* value noted<br>*Give P values as exact values whenever suitable.*
☒	☐	For Bayesian analysis, information on the choice of priors and Markov chain Monte Carlo settings
☒	☐	For hierarchical and complex designs, identification of the appropriate level for tests and full reporting of outcomes
☒	☐	Estimates of effect sizes (e.g. Cohen's *d*, Pearson's *r*), indicating how they were calculated
☐	☒	Clearly defined error bars<br>*State explicitly what error bars represent (e.g. SD, SE, CI)*

Our web collection on statistics for biologists may be useful.

Software and code

Policy information about availability of computer code

Data collection	Sequencing data were collected using Illumina Mi-Seq at the Centre for Genomic Research, University of Liverpool. Soil mesofauna and property data were collected using standard lab protocols. Soil texture data was collected by LS320 13 analyser from Beckman-Coulter.
Data analysis	Sequence data were trimmed using Cutadapt. OTU tables were constructed from trimmed sequences using a combination of USEARCH 7.0 and VSEARCH 2.3.2 software. OTU tables were analysed in R 3.3.3 using the phyloseq package. Statistical analyses were also conducted in R using the vegan, pls, nlme, and AICcmodavg packages.

For manuscripts utilizing custom algorithms or software that are central to the research but not yet described in published literature, software must be made available to editors/reviewers upon request. We strongly encourage code deposition in a community repository (e.g. GitHub). See the Nature Research guidelines for submitting code & software for further information.

April 2018

Data

Policy information about availability of data

All manuscripts must include a data availability statement. This statement should provide the following information, where applicable:

- Accession codes, unique identifiers, or web links for publicly available datasets
- A list of figures that have associated raw data
- A description of any restrictions on data availability

Data associated with this paper will be publically published in the National Environment Research Council (NERC) Environmental Information Data Centre (EIDC) but are currently under embargo by the Welsh Government. Sequences with limited sample metadata have been uploaded to The European Nucleotide Archive and can be accessed with the following primary accession codes after the end of data embargo (27 June, 2020): PRJEB27883 (16S), PRJEB28028 (ITS), and PRJEB28067 (18S). Data are also available from the authors upon reasonable request with permission from the Welsh Government. The source data underlying Fig. 3a-e is provided as a Source Data file.

Field-specific reporting

Please select the best fit for your research. If you are not sure, read the appropriate sections before making your selection.

☐ Life sciences ☐ Behavioural & social sciences ☒ Ecological, evolutionary & environmental sciences

For a reference copy of the document with all sections, see nature.com/authors/policies/ReportingSummary-flat.pdf

Ecological, evolutionary & environmental sciences study design

All studies must disclose on these points even when the disclosure is negative.

Study description	The data presented here was collected as part of the Glastir Monitoring & Evaluation Programme (https://gmep.wales/). We evaluated the microbial communities from across Wales collected from plots within elements of the 1 square-kilometre sampling grid of Wales. Samples were categorised by land use as classified based on the methodology of Bunce et al. (doi: 10.5285/5f0605e4-aa2a-48ab-b47c-bf5510823e8f), as well as loss-on-ignition class (based on Emmett et al. 2008. Countryside Survey: Soils report from 2007) and soil type from the National Soil Map and Soil Classification (Cranfield Soil and Agrifood Institute, 2004). However, only results based on land use are presented here. In total, 436 samples were used. This is described in full in the Glastir Monitoring & Evaluation Programme First Year Annual Report to Welsh Government (https://gmep.wales/resources).
Research sample	Samples at each site consisted of 3 co-located soil cores. This included a core for characterising soil properties (texture, chemical analyses), a core from which mesofauna were extracted, and a core from which environmental DNA was extracted.
Sampling strategy	Samples were selected from across the 1 square-kilometre square grid of Wales. This included samples from the Wider Wales Component that were randomly sampled within the strata of Bunce et al.'s (doi: 10.5285/5f0605e4-aa2a-48ab-b47c-bf5510823e8f) Land Classification of Great Britain. This was done to allow for an adoption of methods from the UK's Countryside Survey (Carey et al. 2008. Countryside Survey: UK results from 2007). A power analysis was conducted to determine a minimum of 45 samples was needed from the Wider Wales Component. In addition, the rest of the samples were selected based on mapping layers of the Glastir Advanced scheme across the 1 square-kilometre grid of Wales. This was done to calculate a "Glastir score". This score was then used to randomly select the Targeted Component samples. This is described in full in the Glastir Monitoring & Evaluation Programme First Year Annual Report to Welsh Government (https://gmep.wales/resources).
Data collection	A total of 13 surveyors were trained to collect soil cores and record aboveground characteristics including land use, habitat, and plant community composition as described in full in the Glastir Monitoring & Evaluation Programme First Year Annual Report to Welsh Government https://gmep.wales/resources). These surveyors then returned samples to the Centre for Ecology & Hydrology, Bangor for processing.
Timing and spatial scale	Sampling took place from late spring (May) to early autumn (November) in 2013 and again in 2014. Sampling was split across years to minimise the chances of adverse climatic conditions (i.e. heavy rain, flood) altering soil properties and to collect a greater number of samples. There was no repeat sampling. Using the sampling protocols outlined above, co-located samples were collected for soil properties, environmental DNA (both to 15 cm depth), and mesofauna identification (8cm depth) from up to 3 subplots within each 1 square-kilometre sampling square from across Wales, UK. This is described in full in the Glastir Monitoring & Evaluation Programme First Year Annual Report to Welsh Government (https://gmep.wales/resources).
Data exclusions	Samples which lacked sufficient meta-data, particularly regarding land use classification, and land uses with insufficient replication were removed from the data set (11 from 447).
Reproducibility	All soil property data analyses were quality-checked using standards for each run of each test (following the Countryside Survey soil maul - http://nora.nerc.ac.uk/id/eprint/5201/). OTU tables were rarefied.
Randomization	Sample sites were selected at random based on procedure described above. All samples were assigned independent barcodes after before analysis. Sequencing was performed on random assemblages from homogenised samples. Samples were sequenced separately by year; for mixed linear models, sampling year was used as a random effect term.

Blinding	All samples were assigned independent barcodes after before analysis. Also, barcoded samples were selected at random for order of DNA extraction and sequencing.

Did the study involve field work? ☒ Yes ☐ No

Field work, collection and transport

Field conditions	Sampling occurred under temperate, European summer conditions
Location	Samples were collected from across Wales, United Kingdom.
Access and import/export	Permission for using private land was sought from the Welsh Government and land owners with an understanding of anonymity for data on land owners. This is described in full in the Glastir Monitoring & Evaluation Programme First Year Annual Report to Welsh Government (Contract reference: C147/2010/11). There was no need to import and export samples across borders.
Disturbance	Land was accessed by foot, land owners were briefed on sampling procedures which were approved by Welsh Government.

Reporting for specific materials, systems and methods

Materials & experimental systems

n/a	Involved in the study
☒	☐ Unique biological materials
☒	☐ Antibodies
☒	☐ Eukaryotic cell lines
☒	☐ Palaeontology
☒	☐ Animals and other organisms
☒	☐ Human research participants

Methods

n/a	Involved in the study
☒	☐ ChIP-seq
☒	☐ Flow cytometry
☒	☐ MRI-based neuroimaging
